# AUDIT^2 – A Clinical Audit of the Alcohol Assessment and Management in the Southern Gambling Service – First Round Results

**DOI:** 10.1192/bjo.2025.10689

**Published:** 2025-06-20

**Authors:** Filipa M.A.A. Teixeira, Julia M.A. Sinclair, Samuel R. Chamberlain, Konstantinos Ioannidis

**Affiliations:** 1 Hampshire and Isle of Wight Healthcare NHS Foundation Trust, Southampton, United Kingdom; 2 Department of Psychiatry, Clinical and Experimental Sciences, Faculty of Medicine, University of Southampton, Southampton, United Kingdom; 3 University Hospital Southampton, Southampton, United Kingdom

## Abstract

**Aims:** The audit aimed to evaluate assessment, intervention, and signposting for alcohol use problems in people with gambling disorder presenting to the Southern Gambling Service (SGS).

**Methods:** The study included ninety-eight patients referred to SGS between the 28 December 2023 to 2 April 2024, who completed initial assessments.

Baseline data were analysed to stratify patients’ alcohol use risk based on their extended Alcohol Use Disorder Identification Test (AUDIT-C) and Estimated Weekly Alcohol Consumption (EWAC) scores, which were collected via a digital pre-assessment tool. Clinical assessment letters were also reviewed to assess documented compliance with National Institute for Health and Care Excellence (CG115) guidelines, the Department of Health and Social Care guidance and the Royal College of Physicians regarding appropriate management according to their risk brackets. Outcomes included: (1) determining if those scoring at least 5 on the extended AUDIT-C received a full AUDIT assessment; (2) if higher risk groups (scores of 5–10) received brief interventions and (3) if those with 11 or more received advice on safe alcohol reduction and signposting to alcohol services.

**Results:** Forty-four full records were examined: 26 [59%] patients scored <5 (AUDIT-C, lower risk), 14 [32%] patients scored 5–10 (higher risk) and 4 [9%] scored at least 11. In the latter category, 100% of patients received a formulation discussing their alcohol use and 75% of them an alcohol-related International Classification of Diseases 11 diagnosis as part of this formulation. While 100% completed the AUDIT-C and EWAC, none completed the full AUDIT. 7% of those in the higher risk category received documented brief interventions. Of the possibly dependent patients, 1 (25%) was signposted, based on documentation, to alcohol services and no patients received documented advice on avoiding an abrupt alcohol cessation.

**Conclusion:** The audit highlighted strengths (such as 100% of patients being screened for alcohol use problems using AUDIT-C and EWAC) but also areas for improvement (e.g. in conducting appropriate advice interventions and signposting to alcohol services, and ensuring these steps are clinically documented). Recommendations for improvement included: (1) adding a full AUDIT screening for those scoring at least 5 in the extended AUDIT-C; (2) upskilling staff in brief intervention advice; (3) developing a regional alcohol services directory for signposting; and (4) providing psychoeducation materials on safe alcohol use. After implementation of recommendations, the audit will be repeated.

